# Targeting F-Box Protein Fbxo3 Attenuates Lung Injury Induced by Ischemia-Reperfusion in Rats

**DOI:** 10.3389/fphar.2019.00583

**Published:** 2019-05-24

**Authors:** Kuei-Yi Hung, Wen-I Liao, Hsin-Ping Pao, Shu-Yu Wu, Kun-Lun Huang, Shi-Jye Chu

**Affiliations:** ^1^ The Graduate Institute of Medical Sciences, National Defense Medical Center, Taipei, Taiwan; ^2^ Department of Emergency Medicine, Tri-Service General Hospital, National Defense Medical Center, Taipei, Taiwan; ^3^ Institute of Aerospace and Undersea Medicine, National Defense Medical Center, Taipei, Taiwan; ^4^ Department of Internal Medicine, Tri-Service General Hospital, National Defense Medical Center, Taipei, Taiwan

**Keywords:** acute lung injury, ischemia-reperfusion, Fbxo3, BC-1215, Fbxl2, tumor necrosis factor receptor-associated factors

## Abstract

**Background:** Increasing evidence suggests that Fbxo3 signaling has an important impact on the pathophysiology of the inflammatory process. Fbxo3 protein inhibition has reduced cytokine-driven inflammation and improved disease severity in animal model of *Pseudomonas*-induced lung injury. However, it remains unclear whether inhibition of Fbxo3 protein provides protection in acute lung injury induced by ischemia-reperfusion (I/R). In this study, we investigated the protective effects of BC-1215 administration, a Fbxo3 inhibitor, on acute lung injury induced by I/R in rats.

**Methods:** Lung I/R injury was induced by ischemia (40 min) followed by reperfusion (60 min). The rats were randomly assigned into one of six experimental groups (*n* = 6 rats/group): the control group, control + BC-1215 (Fbxo3 inhibitor, 0.5 mg/kg) group, I/R group, or I/R + BC-1215 (0.1, 0.25, 0.5 mg/kg) groups. The effects of BC-1215 on human alveolar epithelial cells subjected to hypoxia-reoxygenation (H/R) were also examined.

**Results:** BC-1215 significantly attenuated I/R-induced lung edema, indicated by a reduced vascular filtration coefficient, wet/dry weight ratio, lung injury scores, and protein levels in bronchoalveolar lavage fluid (BALF). Oxidative stress and the level of inflammatory cytokines in BALF were also significantly reduced following administration of BC-1215. Additionally, BC-1215 mitigated I/R-stimulated apoptosis, NF-κB, and mitogen-activated protein kinase activation in the injured lung tissue. BC-1215 increased Fbxl2 protein expression and suppressed Fbxo3 and TNFR associated factor (TRAF)1–6 protein expression. BC-1215 also inhibited IL-8 production and NF-κB activation *in vitro* in experiments with alveolar epithelial cells exposed to H/R.

**Conclusions:** Our findings demonstrated that Fbxo3 inhibition may represent a novel therapeutic approach for I/R-induced lung injury, with beneficial effects due to destabilizing TRAF proteins.

## Introduction

Tumor necrosis factor receptor (TNF-R)-associated factors (TRAF) represent intracellular signaling adaptor proteins involving a wide range of biological functions (Xie, 2013). TRAF proteins 1–7 (TRAF1–7) are mediators between a wide array of cell surface receptors and downstream signaling events in the regulation of inflammatory reactions, innate and adaptive immune responses, stress reactions, and apoptosis (Xie, 2013; Lalani et al., 2018). The existence of TRAF7 is controversial because the protein without the TRAF homology domain is not similar to other members of the TRAF family. Furthermore, TRAF-mediated signaling pathways typically trigger the activation of nuclear factor-κB (NF-κB) and mitogen-activated protein kinases (MAPKs), which have been demonstrated to tightly mediate the inflammatory response (Shi and Sun, 2018). TRAF-mediated cytokine production *via* NF-κB and MAPK pathways can cause severe tissue edema and injury, multiorgan failure, and shock (Shi and Sun, 2018). Therefore, TRAF proteins have a crucial role in mediating signal transduction to prompt activation of downstream proinflammatory cytokines, greatly impacting host reactions. These findings suggest that targeting inhibition of TRAFs may provide a novel therapeutic strategy in various inflammatory diseases (Lalani et al., 2018).

Protein ubiquitination is an essential post-translational modification that guides proteins for degradation *via* proteasomes or by lysosomes to control intracellular signaling events. Under normal conditions, an ubiquitin E3 ligase that is part of the Skp1-Cullin1-F-box (SCF) family containing F-box and leucine-rich repeat protein 2 (Fbxl2) polyubiquitinates and subsequently degrades TRAF1–6 proteins to limit inflammatory cytokines levels (Chen et al., 2013). By contrast, under inflammatory conditions, Fbxl2 becomes phosphorylated, and a second SCF E3 ligase, with F-box only protein 3 (Fbxo3), destabilizes the sentinel TRAF inhibitor Fbxl2, thereby promoting TRAF1–6 signaling and cytokine gene transcription (Chen et al., 2013). Furthermore, subjects with sepsis had significantly more Fbxo3 protein and less immunoreactive Fbxl2 protein in circulating white blood cells (Chen et al., 2013). Fbxo3 has a 125-amino-acid ApaG domain in its C terminus, which was required for ubiquitination of Fbxl2. A small-molecule Fbxo3 inhibitor, BC-1215, interacted with the Fbxo3–ApaG domain, and it exhibited a low IC_50_ and a high LC_50_ *in vitro* (Chen et al., 2013; Mallampalli et al., 2013). BC-1215 decreased Fbxo3-Fbxl2 interaction, prevented SCF-Fbxo3-catalyzed Fbxl2 ubiquitination, and then lowered amounts of TRAF1–6 proteins (Chen et al., 2013; Mallampalli et al., 2013). Administration of BC-1215 that destabilizes TRAF1–6 protein levels sufficiently ameliorated cytokine-driven inflammation in lipopolysaccharides-induced sepsis, carrageenan-induced paw edema, H1N1 influenza-induced lung injury, dextran sulfate sodium-induced colitis, and tetradecanoylphorbol acetate-induced ear edema (Chen et al., 2013; Mallampalli et al., 2013).

Knowledge of Fbxo3 proteins in inflammatory lung disease is limited. Administration of BC-1215, an Fbxo3 inhibitor, has been shown to increase survival and improve lung injury in H1N1 influenza-infected mice (Chen et al., 2013). BC-1215 or Fbxo3 knockdown attenuated *Pseudomonas aeruginosa* and H1N1 influenza-induced lung injury (Chen et al., 2013; Mallampalli et al., 2013). Administration of BC-1215 also reduced the lavage proinflammatory cytokine levels, protein concentration and cell counts, decreased cell infiltrates in lung tissue, and prolonged survival in a mouse model of cecal ligation and puncture (CLP)-induced sepsis (Chen et al., 2013).

Ischemia-reperfusion (I/R) injury continues to be a primary reason for early primary graft dysfunction and failure after lung transplantation. Despite the advanced care and therapy of critically ill patients, I/R-induced acute lung injury (ALI) remains associated with significant morbidity and poor outcomes (De Perrot et al., 2003; Thompson et al., 2017). I/R injury represents a complicated event with multiple overlapping pathways. Therefore, more efforts are necessary to elucidate the underlying molecular mechanisms of I/R-induced ALI and to develop novel effective therapies. The role of Fbxo3 during I/R-induced ALI is not well understood; hence, the current study investigated whether Fbxo3 inhibition suppressed the development of I/R-induced ALI using a highly selective small-molecule Fbxo3 antagonist, BC-1215.

## Materials and Methods

### Preparation of Isolated Perfused Rat Lungs

The care of rats in this study was conducted in accordance with the Guide for the Care and Use of Laboratory Animals. The experimental protocol was approved by the Animal Review Committee of National Defense Medical Center, Taipei, Taiwan. Isolated perfused rat lungs were established as previously described by our laboratory (Chu et al., 2002; Wu et al., 2015; Liao et al., 2017). Briefly, Sprague-Dawley male rats (350 ± 20 g) were ventilated with 21% O_2_–5% CO_2_ at a rate of 60 breaths/min, a tidal volume of 3 ml, and a positive end-expiratory pressure of 1 cm H_2_O. After a median sternotomy, heparin (1 U/g body weight, [BW]) was injected into the right ventricle, and 10 mL of blood was collected from the right ventricle. The cannulas were placed in the pulmonary artery and left ventricle. The lung was perfused at a constant flow rate (8–10 ml/min) using a physiological salt solution (119 mM NaCl, 4.7 mM KCl, 1.17 mM MgSO_4_, 22.6 mM NaHCO_3_, 1.18 mM KH_2_PO_4_, 1.6 mM CaCl_2_, 5.5 mM glucose, and 50 mM sucrose) containing 4% bovine serum albumin. A total of 10 ml collected blood was added to the perfusate as a “half-blood” solution before recirculation. The recirculating perfusate with the isolated lungs *in situ* was placed on an electronic balance to record real-time changes in lung weight (LW). Pulmonary venous pressure (PVP) and pulmonary arterial pressure (PAP) were continuously monitored from the side arm of the cannula using pressure transducers.

### Experimental Design

The rats were randomized into the following six groups: normal saline group (control, *n* = 6), BC-1215 group (0.5 mg/kg, drug control, *n* = 6), I/R group (*n* = 6), or I/R with different doses of BC-1215 group (0.1 mg, 0.25 mg, or 0.5 mg/kg; *n* = 6 per group). BC-1215 (Sigma-Aldrich, USA) was added to the reservoir (containing 20 mL of perfusate). BC-1215 dose was selected based on previous investigations (Chen et al., 2013; Mallampalli et al., 2013). For induction of I/R, the lungs were stopped ventilation and perfusion to cause ischemia and maintained in the deflated state for 40 min. Then, perfusion and ventilation were resumed for 60 min.

### Measurement of Capillary Permeability

The capillary permeability (K_f_) was calculated using the change in lung weight triggered by the elevation of venous pressure as described previously (Li et al., 2009; Wu et al., 2015; Liao et al., 2017). K_f_ was expressed as milliliters per minute per centimeter water per 100 g lung tissue.

### The Levels of Lung Weight/Body Weight and Wet/Dry (W/D) Weight Ratios

The right lung was excised in the hilar region following the experimental protocol. The wet lung weight was determined, and the LW/BW ratio was calculated. A part of the right upper lung lobe was put in an oven for 48 h at 60°C to obtain the dry weight, and the W/D weight ratio was assessed.

### Bronchoalveolar Lavage Analysis of Protein Content, Cytokine, and Total Cell Counts

The bronchoalveolar lavage fluid (BALF) was obtained by lavaging the left lung (2×) with 2.5 ml of saline after the experiment. The BALF was centrifuged at 200 × *g* for 10 min at room temperature. BALF protein concentration was measured using a bicinchoninic acid protein assay kit (Pierce, Rockford, IL, USA). BALF tumor necrosis factor-α (TNF-α, catalog number: RTA00), cytokine-induced neutrophil chemoattractant-1(CINC-1, catalog number: RCN100, also termed chemokine (C-X-C motif) ligand 1 [CXCL1]), and interleukin-6 (IL-6, catalog number: R6000B) levels were determined using a commercial rat ELISA kit (R&D Systems Inc., Minneapolis, MN, USA). BALF total cell counts were assessed as described previously (Wu et al., 2015).

### Detections of Protein Carbonyl Contents and Malondialdehyde (MDA) Levels in Lung Tissue

MDA was estimated as previously described (Liao et al., 2017; Wu et al., 2017). Briefly, after reaction of MDA with thiobarbituric acid reactive substances, the reaction product was measured (absorbance: 532 nm) and expressed as nmol/mg protein. Protein oxidation in lung tissue was assessed using an assay that measured protein carbonyl content through the interaction of the supernatant with dinitrophenylhydrazine as previously described (Liao et al., 2017; Wu et al., 2017). Results were represented as protein carbonyl content (nmol/mg protein).

### Western Blotting Analysis

Lung tissue homogenate and cell protein lysates (30 μg/lane) were separated using 10–12% sodium dodecyl sulfate-polyacrylamide gel electrophoresis, and immunoblots were developed as previously described (Wu et al., 2015; Liao et al., 2017). The blots were probed with primary antibodies against TRAF1 (catalog number: RG2234242C), TRAF2 (catalog number: RG2234515), Fbxo3 (catalog number: RF22253318), Fbxl2 (catalog number: RF2225661H; 1:500, Thermo Fisher Scientific, Rockford, IL, USA), TRAF3 (catalog number: TA322871), and TRAF5 (catalog number: TA323403; 1:500, OriGene Technologies, Rockville, MD, USA), TRAF4 (catalog number: OAAF07255-10; 1:500, Avivasysbio, San Diego, CA, USA), TRAF6 (catalog number: SC-8409; 1:500, Santa Cruz Biotechnology, Dallas, TX, USA), B-cell lymphoma (Bcl)-2 (catalog number: SC-7382; 1:200, Santa Cruz Biotechnology, Dallas, TX, USA), NF-κB p65 (catalog number: 8242), phospho-NF-κB p65 (catalog number: 3033), inhibitor of NF-κB (IκB)-α (catalog number: 4812), extracellular signal-related protein kinase 1/2 (ERK1/2) (catalog number: 9102), phosho-ERK1/2 (catalog number: 9106), c-Jun N-terminal kinase (JNK; catalog number: 9252), phospho-JNK (catalog number: 9251), p38 protein kinase (p38; catalog number: 9228), phospho-p38 (catalog number: 4511) and cleaved caspase-3 (catalog number: 9664), proliferating cell nuclear antigen (PCNA) (catalog number: 2586; 1:1000, Cell Signaling Technology, Danvers, MA, USA), and β-actin (catalog number: A5441; 1:10000, Sigma Chemical Company, St. Louis, MO, USA). Results were presented as the relative ratio of the target protein to the reference protein.

### Immunohistochemical Analyses

Immunohistochemical staining for myeloperoxidase (MPO) was performed as described previously (Liao et al., 2017; Wu et al., 2017). Briefly, paraffin-embedded lung tissue sections were deparaffinized, then immersed in 3% H_2_O_2_ and 100% methanol for 15 min to block endogenous peroxidase. A rabbit polyclonal antibody to MPO (catalog number: RB-373-A0, 1:100, Thermo Fisher Scientific, Rockford, IL, USA) was used for immunostaining lung sections. After three washes with PBS (5 min each), the slides were incubated with rat-specific horseradish peroxidase polymer anti-rabbit antibody (Nichirei Corporation, Tokyo, Japan) for 30 min. Following three rinses with PBS (5 min each), horseradish peroxidase substrate was added, and the reaction was allowed to occur for 3 min. The tissue sections were then counterstained using hematoxylin. To prove the staining results, all tests were performed in triplicate.

### Histopathology

Lung tissue sections were stained with hematoxylin and eosin. The number of polymorphonuclear neutrophils in the interstitium was determined by counting the number of cells in 10 high-power fields (×400) and averaged. Two investigators who were blinded to the slide sources examined a minimum of 10 randomly selected fields. Semiquantitative grading of the extent of pathological lesions on hematoxylin and eosin sections was performed as previously described (Wu et al., 2015).

### Induction of Hypoxia-Reoxygenation (H/R) in A549 Cells

Human type II alveolar epithelial cells (A549) were obtained from the Food Industry Research and Development Institute (BCRC 60074, Hsinchu, Taiwan). Cells were cultured in RPMI-1640 medium containing 10% fetal bovine serum (Hyclone), penicillin, and streptomycin under 5% CO_2_-95% air humidity at 37°C. A549 cells were challenged under hypoxic conditions for 24 h (1% O_2_-5% CO_2_-94% N_2_) followed by 4 h of reoxygenation (5% CO_2_-95% air; Wu et al., 2013, 2017; Liao et al., 2017). The vehicle or BC-1215 (10 μg/ml) was added before hypoxia. The control group was incubated under normoxic conditions. The cultured supernatant was harvested for measurement of IL-8 using ELISA (catalog number: DY208-05, R&D, Inc., Minneapolis, MN, USA).

### Data Analysis

All data analyses were performed using GraphPad Prism 5 statistical software (GraphPad Software, San Diego, CA, USA). Results are expressed as means ± SD. Multiple comparisons among the groups were analyzed using a one-way ANOVA followed by a *post-hoc* Bonferroni test. Two-way ANOVA for repeated measurements followed by the *post-hoc* Bonferroni test was conducted for comparisons of lung weight gain and PAP between groups. Significance was defined as *p* < 0.05.

## Results

### BC-1215 Improves Pulmonary Microvascular Barrier Function and Attenuates PAP Elevation Induced by I/R

**Figure 1:**
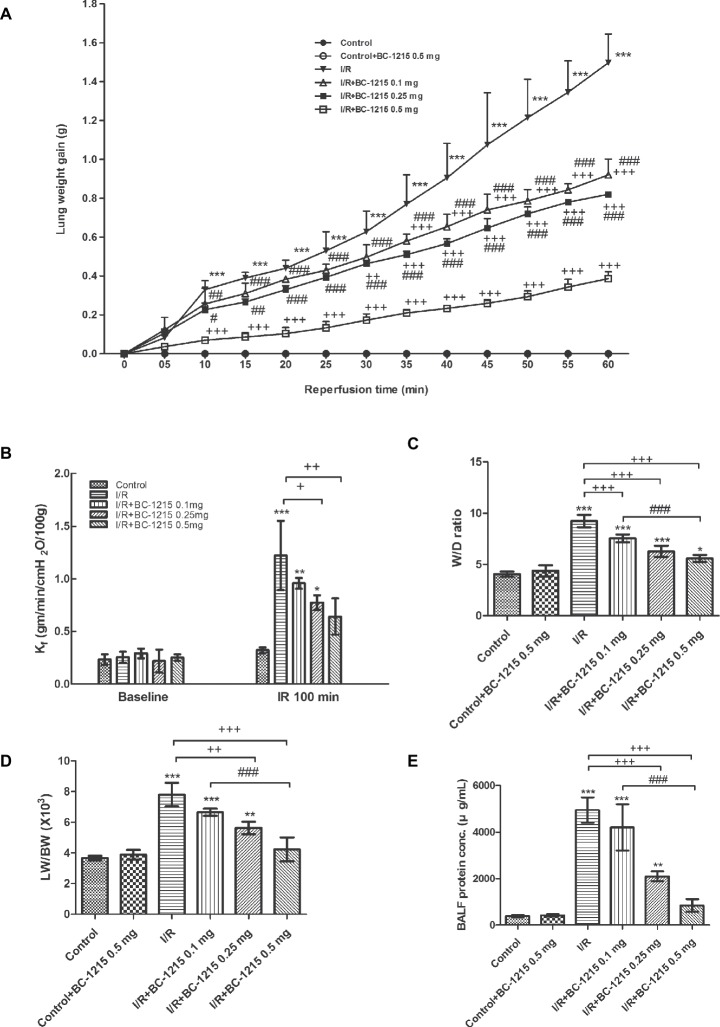
Effect of BC-1215 on lung edema. Lung weight gain **(A)**, K_f_ **(B)**, lung wet/dry (W/D), weight ratio **(C)**, lung weight/body weight (LW/BW) ratio **(D)**, and protein concentrations in bronchoalveolar lavage fluid (BALF) **(E)** significantly increased in the ischemia-reperfusion (I/R) group. Treatment with BC-1215 significantly attenuated the increase in these parameters. Data are expressed as mean ± SD (*n* = 6 per group). ^*^p < 0.05, ^**^p < 0.01, ^***^p < 0.001 compared with the control group; ^+^*p* < 0.05, ^++^*p* < 0.01, ^+++^*p* < 0.001 compared with the I/R group; ^#^*p* < 0.05, ^##^*p* < 0.01 ^###^*p* < 0.001 compared with the I/R + BC-1215 0.5 mg group.

Treatment with BC-1215 attenuated the increase in lung weight gain associated with I/R (Figure 1A). In addition, I/R significantly induced an increase in K_f_, W/D weight and LW/BW ratios, and BALF protein concentration (*p* < 0.05; Figures 1B–E); BC-1215 treatment significantly ameliorated these increases in a dose-dependent manner.

**Figure 2:**
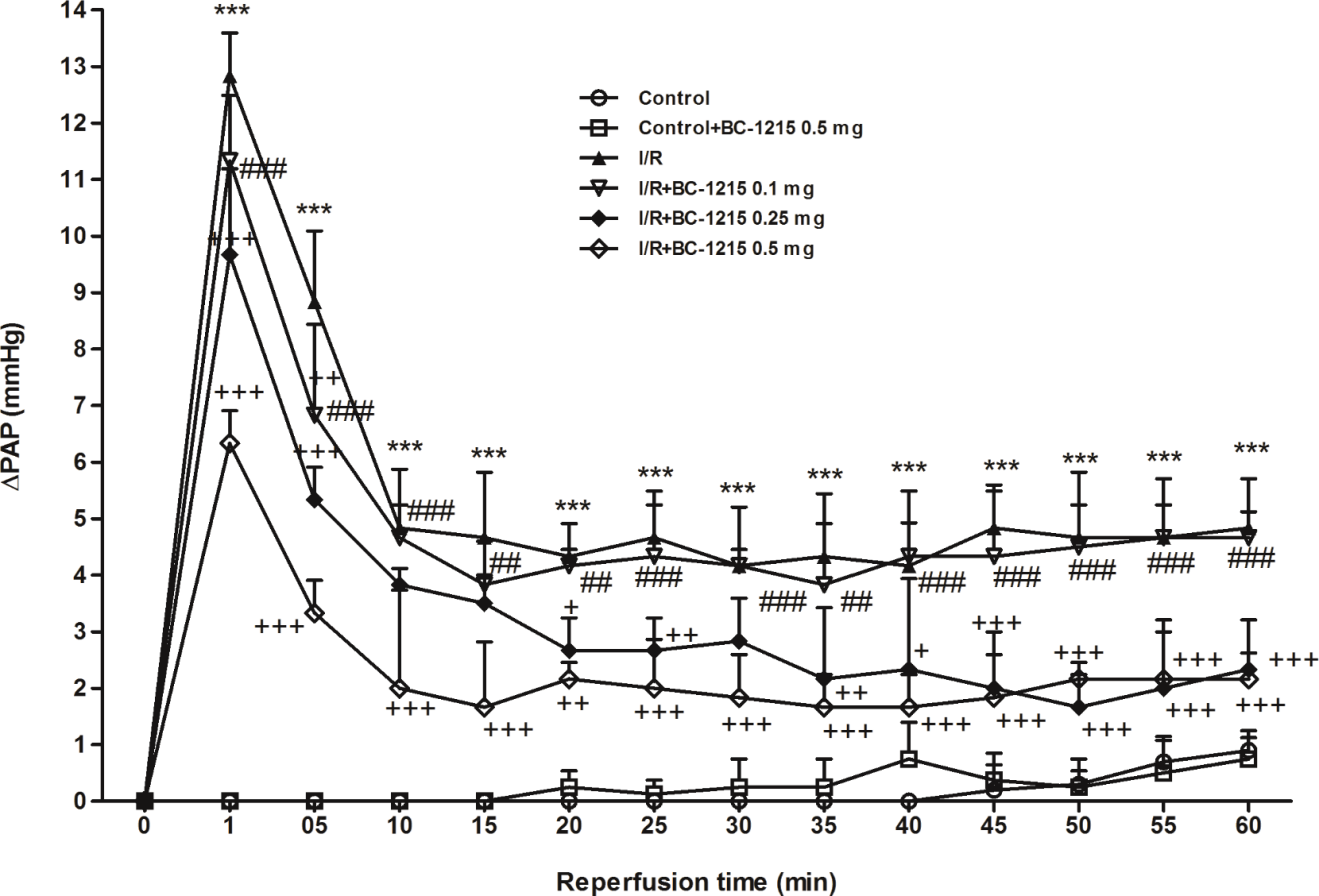
Effect of BC-1215 on pulmonary artery pressure (ΔPAP). PAP increased significantly in the ischemia-reperfusion (I/R) group. The increase in PAP was attenuated significantly following the treatment with BC-1215. Data are expressed as mean ± SD (*n* = 6 per group). ***p < 0.001, compared with the control group; ^+^*p* < 0.05, ^++^*p* < 0.01, ^+++^*p* < 0.001 compared with the I/R group; ^##^*p* < 0.01, ^###^*p* < 0.001 compared with the I/R + BC-1215 0.5 mg group.

PAP kept constant in the control group for 100 min during the observation period. PAP initially increased in the I/R group and then dropped after reperfusion. After 60 min of reperfusion, PAP was significantly elevated in the I/R group compared with baseline values and compared with controls. BC-1215 treatment significantly reduced the elevation observed in PAP in a dose-dependent manner (*p* < 0.05; Figure 2).

### BC-1215 Inhibits Fbxo3 and TRAF1–6 but Increases Fbxl2 Protein Expression in I/R Lung Tissue

**Figure 3:**
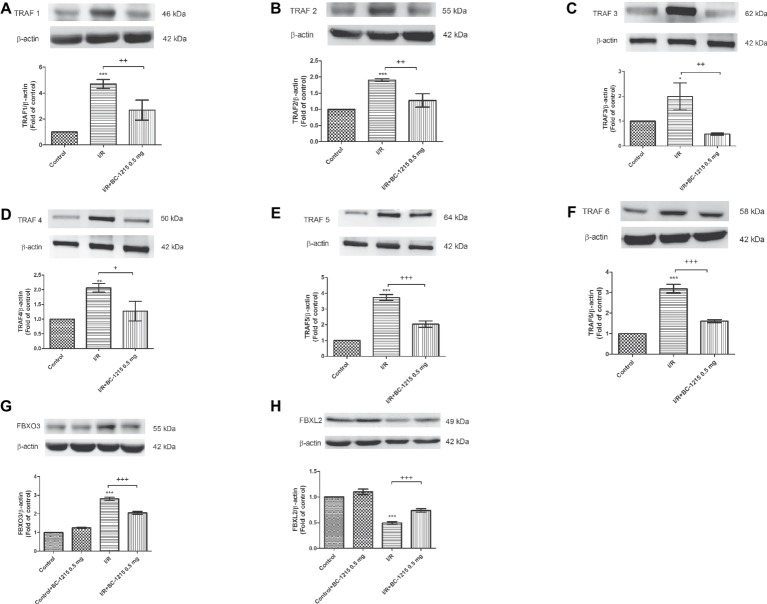
Effect of BC-1215 on Fbxl2, Fbxo3, and TRAF1–6 protein expressions in lung tissue. Western blot and densitometry analysis of TRAF1 **(A)**, TRAF2 **(B)**, TRAF3 **(C)**, TRAF4 **(D)**, TRAF5 **(E)**, TRAF6 **(F)**, Fbxo3 **(G)**, and Fbxl2 **(H)** protein in the lung tissue. β-actin served as loading control for cytoplasmic proteins. Representative blots are shown. Ischemia-reperfusion (I/R) significantly increased protein expression of TRAF1–6 and Fbxo3 and decreased Fbxl2 protein expression in lung tissue compared with the control group. BC-1215 significantly decreased the degree of TRAF1-6 and Fbxo3 protein expression and increased Fbxl2 protein expression in the I/R group. Data are expressed as mean ± SD (*n* = 3 per group). *p < 0.05, **p < 0.01, ***p < 0.001 compared with the control group; ^+^*p* < 0.05, ^++^*p* < 0.01, ^+++^*p* < 0.001 compared with the I/R group.

Control animals weakly expressed TRAF1–6 and Fbxo3, whereas lung tissues strongly expressed these proteins after I/R injury. BC-1215 treatment effectively lowered the amounts of TRAF1–6 and Fbxo3 proteins in the lungs (Figures 3A–G). Furthermore, I/R significantly decreased Fbxl2 protein expression compared with the control group (*p* < 0.05; Figure 3H). BC-1215 treatment significantly increased Fbxl2 protein expression in the I/R group.

### BC-1215 Attenuates Expression of Proinflammatory Cytokines and Total Cell Counts in the BALF After I/R

**Figure 4:**
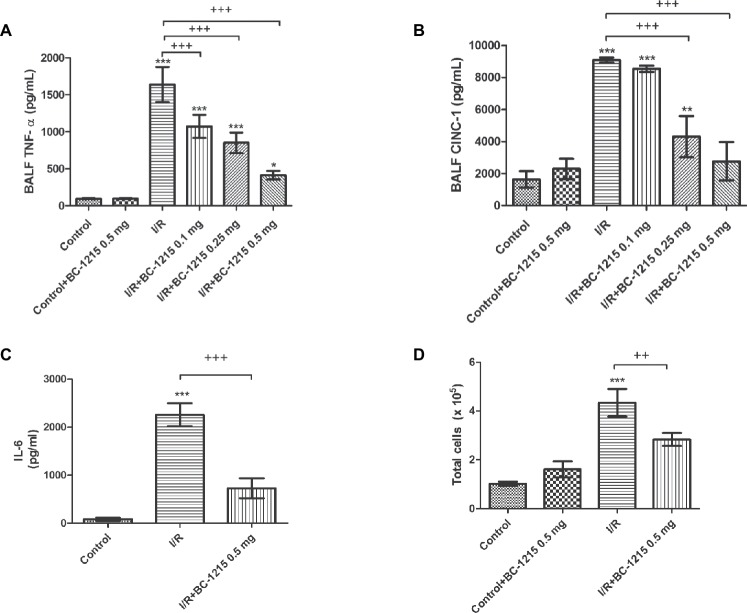
Effect of BC-1215 on TNF-α, CINC-1, IL-6 levels, and total cell counts in bronchoalveolar lavage fluid (BALF). TNF-α **(A)**, CINC-1 **(B)** and IL-6 **(C)** levels and total cell counts **(D)** in BALF were significantly increased in the ischemia-reperfusion (I/R) group. Treatment with BC-1215 significantly attenuated these increases in BALF. Data are expressed as mean ± SD (*n* = 6 per group). ^*^p < 0.05, ^**^p < 0.01, ^***^p < 0.001 compared with the control group; ^++^*p* < 0.01, ^+++^*p* < 0.001 compared with the I/R group.

Levels of TNF-α, CINC-1, and IL-6 and total cell counts were significantly increased in BALF from the I/R group compared with controls (*p* < 0.05; Figure 4). BC-1215 significantly inhibited the I/R-induced increase of TNF-α, CINC-1, IL-6, and total cell counts in BALF (*p* < 0.05; Figure 4).

### BC-1215 Decreases Carbonyl Content, Malondialdehyde Level, and MPO-Positive Cells in I/R Lung Tissue

**Figure 5:**
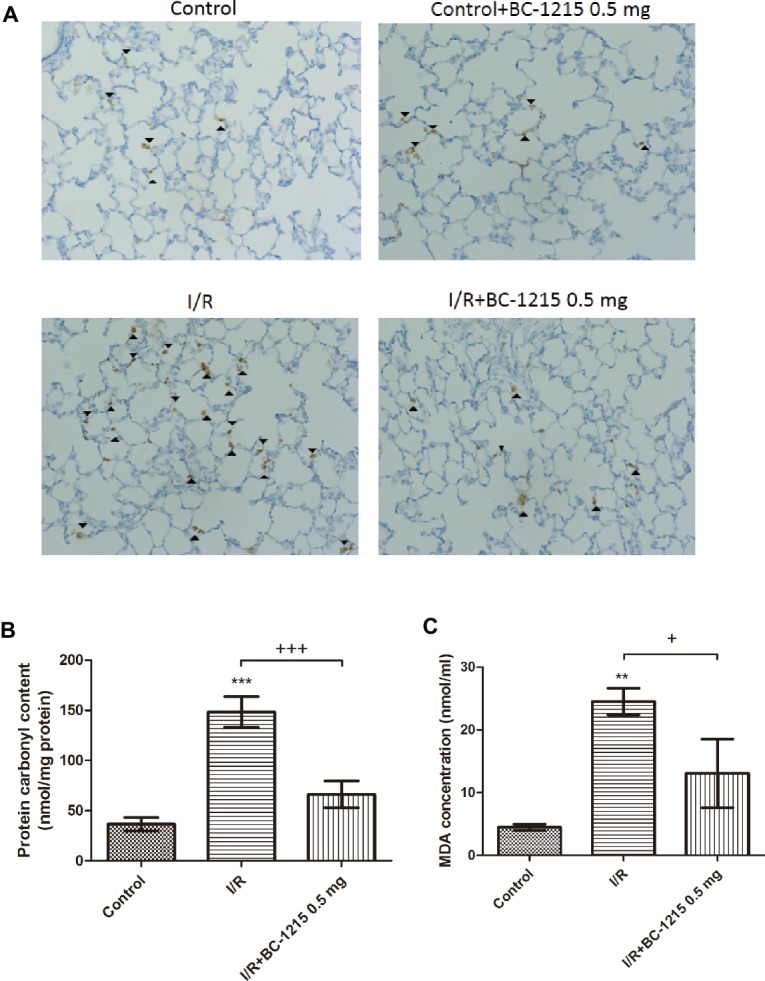
Effect of BC-1215 on protein carbonyl content, MDA levels, and MPO-positive cells in lung tissue. MPO-positive cells **(A)**, carbonyl contents **(B)**, and MDA levels **(C)** in lung tissue were significantly increased in the ischemia-reperfusion (I/R) group. BC-1215 treatment significantly attenuated these increases. **A**, Immunohistochemistry for MPO in the lung (indicated with arrowhead) (200× magnification). Data are expressed as mean ± SD (*n* = 6 per group). ^**^p < 0.01, ^***^p < 0.001 compared with the control group; ^+^*p* < 0.05, ^+++^*p* < 0.001 compared with the I/R group.

The I/R group had significantly increased levels of malondialdehyde, carbonyl content, and MPO-positive cells in lung tissue compared with the control group (*p* < 0.05; Figures 5A–C). Treatment with BC-1215 significantly mitigated these observed increases.

### Neutrophil Infiltration and Histological Changes After I/R Are Attenuatedby BC-1215

**Figure 6:**
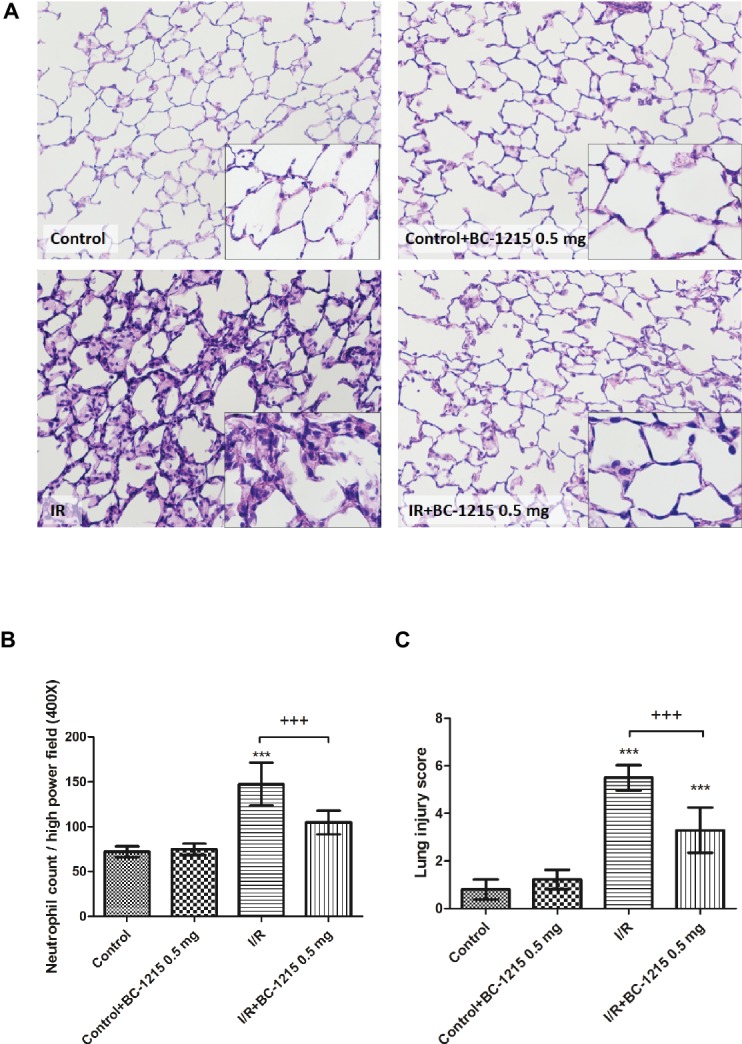
Effect of BC-1215 on lung pathology. As shown by a representative micrograph of lung tissue (200× magnification) **(A)**, neutrophil infiltration and septal edema were increased in the ischemia-reperfusion (I/R) group. BC-1215 treatment significantly attenuated these histopathological changes, the numbers of neutrophils per high power field (400× magnification) **(B)**, and the lung injury scores **(C)**. Data are expressed as mean ± SD (*n* = 6 per group). *** *p* < 0.001, compared with the control group; ^+++^ *p* < 0.001, compared with the I/R group.

Histological analysis revealed a significantly higher interstitial thickening and cellular infiltration in the I/R group compared with the control group (Figure 6A). Treatment with BC-1215 significantly diminished histological changes, neutrophil infiltration (Figure 6B), and lung injury scores (Figure 6C) in the I/R group.

### BC-1215 Increases Bcl-2 but Reduces Caspase-3 Protein Expression in I/R Lung Tissue

**Figure 7:**
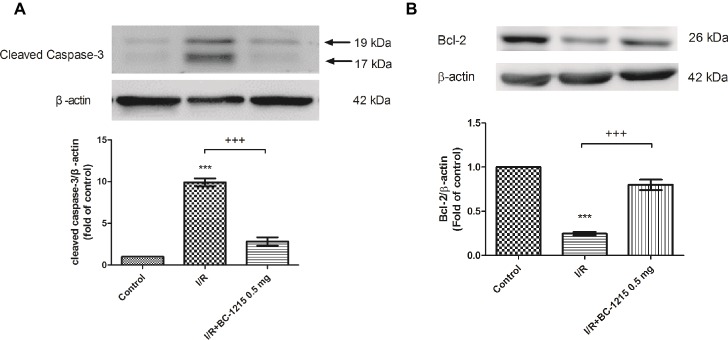
Effect of BC-1215 on the expression of caspase-3 and Bcl-2 in lung tissue. Western blot analysis of caspase-3 **(A)** and Bcl-2 **(B)** protein in the lung tissue. β-actin served as a loading control for cytoplasmic proteins. Representative blots are shown. Ischemia-reperfusion (I/R) significantly decreased Bcl-2 protein expression and induced caspase-3 expression in the lung tissue. BC-1215 treatment significantly increased Bcl-2 protein expression and decreased caspase-3 protein expression in the I/R group. Representative blots are shown. Data are expressed as mean ± SD (*n* = 3 per group). ***p < 0.001, compared with the control group; ^+++^*p* < 0.001, compared with the I/R group.

Bcl-2 protein expression in lung tissue was significantly reduced in the I/R groups compared with the control group but was significantly increased upon administration of BC-1215 (Figure 7A). Caspase-3 protein was significantly increased in the I/R group compared with the control group, but BC-1215 treatment significantly attenuated the observed increase (Figure 7B).

### BC-1215 Attenuates the Mitogen-Activated Protein Kinase (MAPK) Signaling Pathway in I/R Lung Tissue

**Figure 8:**
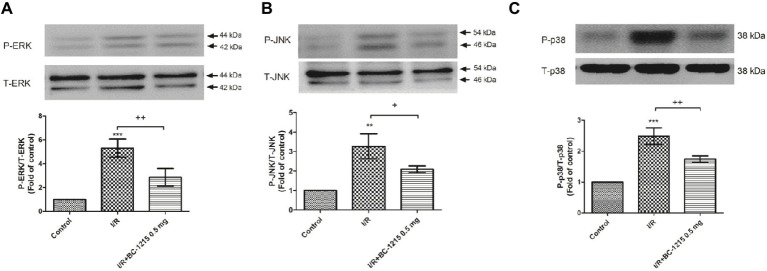
Effect of BC-1215 on mitogen-activated protein kinase (MAPK) expressions in lung tissue. The phosphorylation of ERK **(A)**, JNK **(B)**, and p38 **(C)** was enhanced in the ischemia-reperfusion (I/R) group. BC-1215 treatment attenuated these effects. A representative blot is shown. All data are shown as mean ± SD (*n* = 3 per group). **p < 0.01, ***p < 0.001, compared with the control group; ^+^*p* < 0.05, ^++^*p* < 0.01, ^+++^*p* < 0.001, compared with the I/R group.

Western blot analyses demonstrated significant increases in the protein amounts of ERK, JNK, and p38 phosphorylation in I/R group. Treatment with BC-1215 significantly attenuated activation of these MAPKs (Figures 8A–C).

### BC-1215 Inhibits the NF-κB Signaling Pathway in I/R Lung Tissue

**Figure 9:**
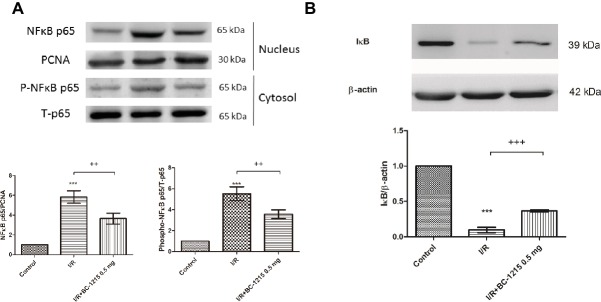
Effect of BC-1215 on NF*-*κB and IκB-α activation in lung tissues. BC-1215 reduced nuclear NF-κB p65 and cytoplasmic phospho-NF-κB p65 levels **(A)** and increased IκB-α levels **(B)** in ischemia-reperfusion (I/R)-induced lung injury. PCNA and β-actin served as loading controls for nuclear and cytoplasmic proteins, respectively. Representative blots are shown. Data are expressed as mean ± SD (*n* = 3 per group). *** *p* < 0.001, compared with the control group;^++^*p* < 0.01, ^+++^*p* < 0.001 compared with the I/R group.

Increased levels of NF-κB p65 in the nucleolus and phospho-NF-κB p65 in cytoplasm were associated with decreased levels of IκB-α in the cytoplasm in the I/R group compared with controls (Figures 9A,B). BC-1215 treatment significantly increased IκB-α levels and reduced nuclear NF-κB p65 and cytoplasmic phospho-NF-κB p65 levels (Figures 9A,B).

### Activation of A549 Cells by H/R is Attenuated by BC-1215

**Figure 10:**
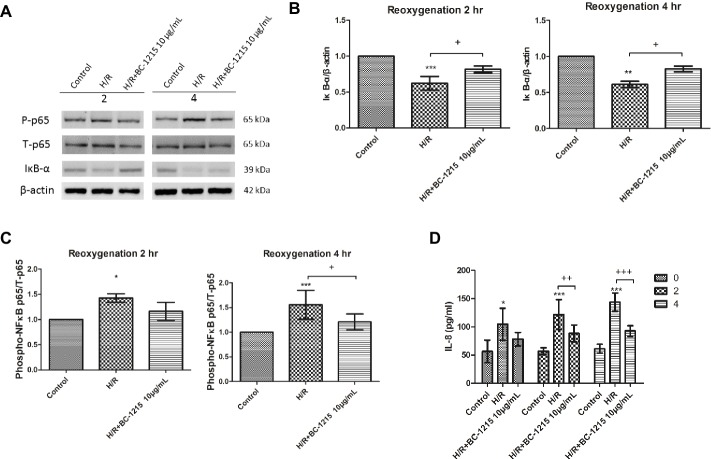
Effect of BC-1215 on A549 cells subjected to hypoxia-reoxygenation (H/R). BC-1215 significantly reduced degradation of IκB-α **(A,B)** at 2 and 4 h, the increase of phosphorylated NF-κB p65 **(A,C)** at 4 h, and IL-8 **(D)** production at 2 and 4 h in A549 cells exposed to H/R. β-actin served as the loading control. A representative blot is shown. Data are expressed as mean ± SD (*n* = 3–6). *p < 0.05, **p < 0.015, ***p < 0.001 compared with the control group; ^+^*p* < 0.05, ^++^*p* < 0.01, ^+++^*p* < 0.001, compared with the H/R group.

Because type II epithelial cells regulate alveolar fluid levels and contribute to host defense and the immune responses, we assessed the effect of BC-1215 on the A549 type II cell line in a model of H/R. The A549 cell line, derived from a human alveolar cell carcinoma, shared many properties of type II alveolar epithelial cell line cells and was utilized because they are the most widely used *in vitro* model for analyzing H/R (Huerter et al., 2016). BC-1215 significantly reduced the increase of phospho-NF-κB p65 protein expression at 4 h and the decrease of IκB-α protein expression at 2 and 4 h after H/R in A549 cells (Figures 10A–C). Furthermore, BC-1215 significantly suppressed levels of IL-8 at 2 and 4 h in the H/R group (Figure 10D).

## Discussion

We currently report that the Fbxo3 inhibitor, BC-1215, successfully protected rats from ALI related to I/R, as evidenced by decreased lung edema, PAP, tissue recruitment of leukocytes, production of inflammatory cytokines, oxidative stress, and apoptosis, as well as the suppression of tissue injury. BC-1215 also significantly decreased TRAF1–6 and Fbxo3 protein content and increased Fbxl2 protein content in rat lung tissue. In addition, BC-1215 inhibited the activation of I/R-induced MAPK and NF-κB pathway. Consistent with the findings in lung tissue from rats, BC-1215 blunted NF-κB signaling activation and IL-8 production observed in A549 epithelial cells subjected to H/R. These results suggest the importance of Fbxo3 in the pathophysiology of I/R-induced lung injury. A treatment that inhibits Fbxo3 may be of value as a novel therapeutic strategy following the reperfusion lung injury after lung transplantation.

Following I/R injury there was a significant increase in Fbxo3 and TRAF1–6 protein expression in lung tissue but a decrease in Fbxl2 expression. This is similar to previously reported results that demonstrated Fbxo3 and TRAF proteins were highly upregulated in a mouse model of dextran sulfate sodium-induced colitis and hepatic and cerebral I/R injury (Chen et al., 2013; Zhang et al., 2014; Hu et al., 2016). TRAFs can be triggered by upstream TNF receptors, Toll-like receptors, and cytokine receptors and promote downstream signaling cascades involved in various biological events, such as cytokine production and cell survival and death (Xie, 2013). TRAFs perform important biological processes in both adaptive and innate immunity (Xie, 2013; Lalani et al., 2018). Recent functional and structural studies have demonstrated that TRAF proteins may have individual functions, and F box protein regulation of this family may lead to divergent biological responses that affect inflammatory response (Chen et al., 2013; Xie, 2013; Lalani et al., 2018). Previous investigations have showed that TRAF1 and TRAF3 contribute to the major inflammatory and apoptotic processes in hepatic I/R (Zhang et al., 2014; Hu et al., 2016). TRAF1 and TRAF5 have been shown to be key mediators in neuronal death and cerebral I/R injury (Lu et al., 2013; Wang et al., 2013). By contrast, TRAF2 and TRAF6 mediate protective signaling in models of myocardial I/R injury (Tzeng et al., 2014; Wang et al., 2014). Furthermore, Fbxl2 targeted TRAF1–6 for ubiquitination and degradation has been shown to decrease cytokine production in inflammatory cells and tissues (Chen et al., 2013). Fbxo3 was as an E3-ubiquitin ligase subunit that induced ubiquitination and degradation of another E3 ligase subunit, Fbxl2, and increased TRAF protein expression (Chen et al., 2013). In the current study, BC-1215 targeted Fbxo3 for ubiquitin-mediated degradation, which enhanced Fbxl2 protein expression and subsequent degradation of TRAF proteins. This finding is consistent with a previous experiment that BC-1215 decreased the expression of Fbxo3 protein in the synaptic plasma membranes of the ipsilateral dorsal horn of mice subjected to spinal nerve ligation (Lai et al., 2016). These observations strongly support a significant role for Fbxo3 in the inflammatory events observed in ALI associated with I/R.

Considerable evidence from clinical investigations and experimental medicine has demonstrated the important role of pro-inflammatory cytokines in the pathogenesis of ALI/ARDS. A complex network of inflammatory cytokines and chemokines has also been shown to mediate, amplify, and perpetuate the lung injury process (Goodman et al., 2003). However, treatments targeting specific anti-cytokines (e.g., anti-TNF-α and IL-8 antibody) for ALI/ARDS mitigated over-exuberant cytokine activity were not successful (Raghavendran et al., 2008). The reason for this may be due to activation of unopposed mediators (Mallampalli et al., 2013). Our experiment showed that inflammatory cytokines such as TNF-α, IL-6, and CINC-1 in BALF were upregulated after I/R-induced lung injury, and BC-1215 significantly suppressed these observed upregulation. Our observations were also similar to those reported in previous studies demonstrating that BC-1215 attenuated cytokine production in *Pseudomonas*-induced lung injury, thereby reducing inflammation (Chen et al., 2013; Mallampalli et al., 2013). Due to the inhibition of TRAF-mediated cytokine production, BC-1215 might afford greater opportunity relative to other agents that target a single cytokine. However, such therapy might be associated with unanticipated and even paradoxical effects, considering immunological surveillance is key in the recognition of foreign pathogens or pre-cancerous and cancerous cells. Strong immunosuppressive therapies are associated with an increased risk of infection and malignancy (Kavanaugh, 2002). Therefore, before the current results can be translated for clinical application, additional preclinical studies are needed to verify these harmful effects.

Investigations over the past several decades have implicated the generation of oxidative/reactive oxygen species in I/R-induced lung injury, specifically their role in modifying cellular proteins, lipids, carbohydrates, and DNA causing epithelial and endothelial damage, which can lead to cell death (Ferrari and Andrade, 2015). The injured vascular endothelial cells will lead to an increase in capillary vascular permeability and efflux of protein-rich fluids into the interstitium, followed by increased neutrophil migration and release of pro-inflammatory cytokines such as TNF-α, IL-6, and IL-8 from activated neutrophils. Our data suggested that BC-1215 reduced production of oxidant/reactive oxygen species as reflected by decreasing protein carbonylation and peroxidation of membrane lipids in I/R lung tissue. In addition, BC-1215 may limit the degree of I/R-evoked neutrophil recruitment into the lung tissue, as demonstrated by a reduction in neutrophils and MPO-positive cells. This reduction prevents interaction between neutrophils and the endothelium and further lowers the levels of proinflammatory cytokines and free radical production by activated neutrophils. Consistent with these observations, BC-1215 significantly inhibited caspase-3 activation and augmented anti-apoptotic protein expression of Bcl-2 after I/R lung injury. Pro-inflammatory cytokines had the ability to initiate the apoptotic cascade through the death receptor/caspase pathway. By contrast, BC-1215 can inhibit pro-inflammatory cytokine production *via* degrading TRAF proteins. Therefore, it is reasonable to speculate that BC-1215 could suppress apoptosis and limit excess cell death responses in I/R-induced ALI. The anti-oxidative, anti-apoptotic, and anti-inflammatory effects of BC-1215 attenuated pulmonary edema as demonstrated by reducing lung weight gain, W/D and LW/BW ratios, K_f_, and protein concentration in BALF.

NF-κB is considered to be a crucial transcription factor that controls pro-inflammatory gene expression during I/R injury. NF-κB is activated by degradation of IκBα in response to pro-inflammatory stimuli and then migrates from the cytoplasm to the nucleus to promote the transcription of the downstream genes. Aberrant activation of the NF-κB pathway has been implicated in the pathophysiology of ALI/ARDS. Our previous and present reports have demonstrated NF-κB activation during lung I/R injury, and the inhibition of its activation during I/R reduces lung injury in rats (Wu et al., 2015, 2017; Liao et al., 2017). It has been recognized that TRAF1, 2, 3, 5, and 6 mediate the activation of NF-κB pathways (Xie, 2013; Zhang et al., 2014; Shi and Sun, 2018). Our results revealed that BC-1215 prevented nuclear translocation of NF-κB and inhibited the degradation of IκΒ in rat lungs exposed to I/R. This is attributed to the hypothesis that BC-1215 inhibits the ability of Fbxo3 to destabilize TRAF proteins. The inhibition of NF-κB activity resulted in decreased production of NF-κB-regulated pro-inflammatory cytokine production (TNF-α, CINC-1, and IL-6). Furthermore, we performed *in vitro* A549 cell culture studies to clarify the effects of BC-1215 on alveolar epithelial cells. Comparable with the observation in rat lung, BC-1215 significantly prevented IκBα degradation and subsequently NF-κB p65 phosphorylation and IL-8 production in A549 cells exposed to H/R simulating the I/R injury. This result indicated that H/R-induced inflammatory response of type II lung alveolar epithelial cells was potentially reversed by BC-1215 *in vitro*.

Activation of MAPK signaling cascades, including p38, ERK, and JNK, contributes to the development of ALI/ARDS. The activated major MAPK subfamilies can induce multiple biological processes, such as immune and inflammatory responses. Previous studies have found that the suppression of p38, ERK, or JNK MAPK effectively inhibited LPS, I/R, and peritonitis-induced lung inflammation (Asaduzzaman et al., 2008; Schuh and Pahl, 2009; Chen et al., 2010; Liao et al., 2017; Wu et al., 2017). TRAF2 and TRAF6 are crucial for activation of the TNFα or IL-1 stimulated MAPK pathway (Xie, 2013; Lalani et al., 2018; Shi and Sun, 2018). In the current study, we reported that I/R induced phosphorylation of p38, ERK, and JNK, and BC-1215 attenuated I/R-induced activation of MAPK *via* TRAF degradation. This may limit extensive inflammation in lung injury associated with I/R. Therefore, the beneficial effects of BC-1215 observed in this study may be partly due to its interference with MAPK signaling. However, the precise molecular mechanisms by which BC-1215 exerts effects in the MAPK signaling pathway require further study.

TRAFs have overlying functions, and individual TRAF molecules (TRAF1–6) also have distinct and imperative functions in inflammatory processes as demonstrated by studies using mouse models with TRAF gene manipulation (Chen et al., 2013; Xie, 2013; Lalani et al., 2018; Shi and Sun, 2018). Further, the role of each specific TRAF molecule in inflammation may modify considerably depending on extracellular stimuli and intracellular molecular processes involved, the specific signal pathways participating, the context of cell and organ, the functional or metabolic state of the cell, and the intracellular TRAF-interacting proteins. By contrast, TRAF2 and TRAF3 have been reported to have anti-inflammatory function that negatively regulates TLR-stimulated expression of pro-inflammatory cytokines (Chen et al., 2013; Xie, 2013; Lalani et al., 2018; Shi and Sun, 2018). Thus, further research to uncover underlying mechanisms that modulate the fate and signaling pathways of TRAFs is needed in the future.

Several limitations are present in this study. First, the isolated lung model may not be extrapolated to the intact whole animal, in which multiple factors were involved. Therefore, *in vivo* studies will be required to verify the role of Fbxo3 protein and its inhibition in I/R-induced lung injury. Second, the doses of BC-1215 in the model were selected based on previous studies (Chen et al., 2013; Mallampalli et al., 2013). The doses of Bc1215 *in vivo* model need further investigations. Third, the molecular evaluations of lungs were performed only after 60 min of reperfusion following 40 min of ischemia. More detailed assessments of the temporal changes in Fbxo3 protein and its inhibition from the ischemic time length *in vivo* model are therefore necessary.

Multiple immune cells are involved in the pathophysiology of I/R injury (Zuidema and Zhang, 2010; Kalogeris et al., 2012). Polymorphonuclear leukocyte cells have been demonstrated to be the major leukocytes found in reperfused tissues following ischemic injury. Neutrophils are considered to be the early cellular mediator for local microvascular changes and parenchymal damage (Kalogeris et al., 2012). Type II alveolar epithelial cells play a pivotal role in the synthesis of pulmonary surfactant, maintaining epithelial barrier stabilization, and immune defense. I/R injury causes the damage in alveolar epithelial cell and leads to the formation of alveolar edema (Whitsett and Alenghat, 2015). Lymphocytes and macrophages penetrate subsequently in I/R injury and likely extend the early injury stage. T cells have been demonstrated to mediate I/R injury in the lung. Recent data suggest that immune cells (CD4, CD8, Th1, Th2, Th17, and regulatory T cells [Tregs]) play as an important role in the complex inflammation of lung injury (Linfert et al., 2009; Lin et al., 2018). The function of Tregs is to stop the development and exacerbation of inflammatory diseases, and they can prevent the development of immune pathology and inflammation involved in ALI (Lin et al., 2018). The role of B cells in I/R injury also has been studied (Kalogeris et al., 2012). Studies using mice deficient in either B cells or components of the complement system, which interact with B-cell receptors have shown that these cells contribute to I/R injury (Linfert et al., 2009). Most evidence from various researches indicates that B lymphocytes are consistently injurious *via* a mechanism involving B cell-derived IgM and activating the complement system (Linfert et al., 2009). However, this current experiment focused on the acute, anti-inflammatory effects of Fbxo3 protein inhibition. It is plausible that Fbxo3 inhibitor may target immune cells and their effects in I/R lung injury. Further investigations are required to clarify this.

In summary, we currently demonstrated that BC-1215, an Fbxo3 protein inhibitor, attenuated lung I/R injury by decreasing lung edema, production of inflammatory cytokines, reactive oxygen species, apoptosis, and NF-κB and MAPK signaling. The protective effect was associated with decreased Fbxo3–Fbxl2 interaction and reduced TRAF protein expressions. Our results suggest that the pharmacological inhibition of Fbxo3 protein and its downstream pathway might offer therapeutic benefit against I/R-induced ALI. However, further understanding of the functional and physiological responses after the use of Fbxo3 protein inhibitors such as BC-1215 is needed before being considered as a therapeutic option in I/R-induced ALI.

## Author Contributions

S-JC and K-LH conceived and designed the experiments. K-YH and W-IL performed the experiments. W-IL, H-PP, and S-YW analyzed the data. S-JC and K-YH wrote the manuscript. All authors read and approved the final manuscript.

### Conflict of Interest Statement

The authors declare that the research was conducted in the absence of any commercial or financial relationships that could be construed as a potential conflict of interest.
